# Retraction Note: LncRNA SNHG7 promotes pancreatic cancer proliferation through ID4 by sponging miR-342-3p

**DOI:** 10.1186/s13578-023-01103-6

**Published:** 2023-08-14

**Authors:** Dongfeng Cheng, Juanjuan Fan, Yang Ma, Yiran Zhou, Kai Qin, Minmin Shi, Jingrui Yang

**Affiliations:** 1 Pancreatic Disease Center, Department of General Surgery, Rui Jin Hospital Affiliated to Shanghai Jiao Tong University School of Medicine, No.197 Rui Jin Er Road, Shanghai, China; 2 Yichuan Community Health, Shanghai, China; 3 Research Institute of Digestive Surgery, Ruijin Hospital, Shanghai Jiaotong University School of Medicine, Shanghai, China

**Retraction note: Cell Biosci (2019) 9:28**

10.1186/s13578-019-0290-2

The Editor-in-Chief has retracted this article because of concerns that a number of panels in Figs. 2d, 4d and 4e are duplicated from previously published articles. Specifically:

- The eight panels in Fig. 2d appear to be the same as panels in Fig. 2B of [1].
- Eleven of the panels in Fig. 4d appear to be the same as panels in Fig. 2C of [1].
- Five of the panels in Fig. 4e appear to overlap with panels in Fig. 3A of [2].

The Editor-in-Chief therefore no longer has confidence in the validity of the data reported. None of the authors has responded to correspondence from the Publisher about this retraction.
